# A machine learning framework for scRNA-seq UMI threshold optimization and accurate classification of cell types

**DOI:** 10.3389/fgene.2022.982019

**Published:** 2022-11-25

**Authors:** Isaac Bishara, Jinfeng Chen, Jason I. Griffiths, Andrea H. Bild, Aritro Nath

**Affiliations:** ^1^ Department of Medical Oncology and Therapeutics, City of Hope Comprehensive Cancer Center, Duarte, CA, United States; ^2^ Irell & Manella Graduate School of Biological Science, City of Hope Comprehensive Cancer Center, Duarte, CA, United States; ^3^ State Key Laboratory of Integrated Management of Pest Insects and Rodents, Institute of Zoology, Chinese Academy of Sciences, Beijing, China

**Keywords:** ScRNA-seq, UMI (unique molecular identifier), QC, quality control, threshold, optimization, gene, cut off

## Abstract

Recent advances in single cell RNA sequencing (scRNA-seq) technologies have been invaluable in the study of the diversity of cancer cells and the tumor microenvironment. While scRNA-seq platforms allow processing of a high number of cells, uneven read quality and technical artifacts hinder the ability to identify and classify biologically relevant cells into correct subtypes. This obstructs the analysis of cancer and normal cell diversity, while rare and low expression cell populations may be lost by setting arbitrary high cutoffs for UMIs when filtering out low quality cells. To address these issues, we have developed a novel machine-learning framework that: 1. Trains cell lineage and subtype classifier using a gold standard dataset validated using marker genes 2. Systematically assess the lowest UMI threshold that can be used in a given dataset to accurately classify cells 3. Assign accurate cell lineage and subtype labels to the lower read depth cells recovered by setting the optimal threshold. We demonstrate the application of this framework in a well-curated scRNA-seq dataset of breast cancer patients and two external datasets. We show that the minimum UMI threshold for the breast cancer dataset could be lowered from the original 1500 to 450, thereby increasing the total number of recovered cells by 49%, while achieving a classification accuracy of >0.9. Our framework provides a roadmap for future scRNA-seq studies to determine optimal UMI threshold and accurately classify cells for downstream analyses.

## Introduction

One of the key objectives in cancer genomics is characterizing the composition and diversity of cancer and normal cells in the tumor microenvironment (TME) (Ren et al., 2018). Several studies have shown that the composition of the TME, such as the prevalence of infiltrating lymphocytes, polarity of myeloid cells and signaling from stromal components play a critical role in the maintenance and progression of malignant cells, and can serve as indicators of therapeutic potential and response (Gooden et al., 2011; Awad et al., 2018; Maibach et al., 2020; Wu et al., 2020; Geng et al., 2021). The study of the TME has been greatly enhanced by the introduction of single cell RNA sequencing (scRNA-seq), which enabled characterizing the diversity and phenotypes of cells in a tumor at a fine resolution (Rubio-Perez et al., 2021; Tang et al., 2022).

Since the introduction of scRNA-seq more than a decade ago, several incremental technological advances have improved the accessibility and quality of transcriptomic analyses (Hwang et al., 2018; Chen et al., 2019). One such advance is the introduction of unique molecular identifiers (UMIs) which allows direct quantification of available transcripts (Islam et al., 2013). While non-UMI scRNA-seq platforms as Smart-Seq2 provide an improved transcript coverage and high level of mappable reads, UMI platforms such as 10X and drop-seq benefit from the limited amplification bias from highly abundant transcripts (Picelli et al., 2014; Zhang et al., 2019). The higher throughput of UMI platforms also improves the detection rates of rare cell populations, such as certain immune cells, within tumor samples (Azizi et al., 2018). Thus, scRNA-seq technologies have greatly enhanced the ability to characterize the diversity of cancer cells and the TME.

However, the ability to accurately classify the cell types in scRNA-seq dataset is often limited by technical factors, such as read quality of the cells. The quality control (QC) process in a typical scRNA-seq pipeline involves identification and filtering out cells of low quality, typically based on the number of UMIs, number of unique genes, and/or the percentage of mitochondrial DNA (mtDNA). The stress induced by droplet-based UMI methods introduces a challenge in ensuring that the UMIs map to healthy cells (Chittur et al., 1988). For example, cells with leaky or damaged membranes can result in a drop in the number of UMIs and genes detected, while the number of UMIs mapping mtDNA may become relatively high (Luecken and Theis, 2019). This complicates the distinction between true low-quality cells and quiescent, small, and/or rare cell populations, thus creating a trade-off between cell quality and diversity during the QC process (Luecken and Theis, 2019).

Since mitochondrial DNA content varies significantly across organisms and tissues, comprehensive analysis of these variables helps to establish universal organism and tissue-specific threshold guidelines (Osorio and Cai, 2021). However, due to the variability in the number of UMI and genes owing to biological and technical factors, a similar universal threshold cannot be established *a priori*. A probabilistic model was proposed to sort out low-quality cells but its accuracy was limited by the prevalence of low-quality cells, which is usually unknown (Hippen et al., 2021). Additionally, several scRNA-seq pre-processing pipelines included different approaches for QC including the option to view the UMI distribution per cell type using user-defined marker genes (McCarthy et al., 2017; Guo et al., 2021; Grandi et al., 2022). However, these approaches generally depend on the user’s judgment to detect outliers (low-quality cells) from reads and/or gene distribution curve. The scRNA-seq literature shows the number of reads threshold selected at QC can vary from as low as 100 and up to 2500 UMIs, yet the rationale for selecting such thresholds is usually missing (Liu et al., 2021; Gambardella et al., 2022; Gao et al., 2022; Karademir et al., 2022; Lian et al., 2022). Another approach which involves an iterative process between the QC step and downstream analysis was also proposed to improve the detection of low-quality cells (Luecken and Theis, 2019). But the mechanism by which the downstream information can be used to optimize an initial reads threshold is not yet defined.

To address the lack of a systematic approach to determine an optimal reads threshold for filtering cells and classifying cells with high accuracy, we have developed a novel machine learning framework that uses cell identity information collected from a high-quality gold standard. Using this approach, we can identify the lowest reads cut-off that can be implemented in an scRNA-seq data and accurately classify cell lineages and subtypes. We used expert-labelled lineage and cell type identities from a gold standard breast cancer scRNA-seq dataset to train the predictive classifiers. We systematically downsampled the reads per cell in the gold standard dataset using a Poisson model and then applied the classifier to predict cell types. We then calculated the prediction accuracies of the classifiers using the known identities of the cells. This allowed us to determine the optimal threshold at which sufficient biological information was retained. Using this approach, we rescued 49% more cells from the gold standard dataset, which is valuable for downstream analyses of the TME. Using two external datasets, we show that our approach can be applied to low expression cells and to subtypes of major cell types as neutrophils and T-cell subtypes, respectively. Importantly, our framework can be extended to any scRNA-seq dataset where users seek to rescue and classify additional cells at optimal read depths.

## Methods

### Analysis workflow

**FIGURE 1:**
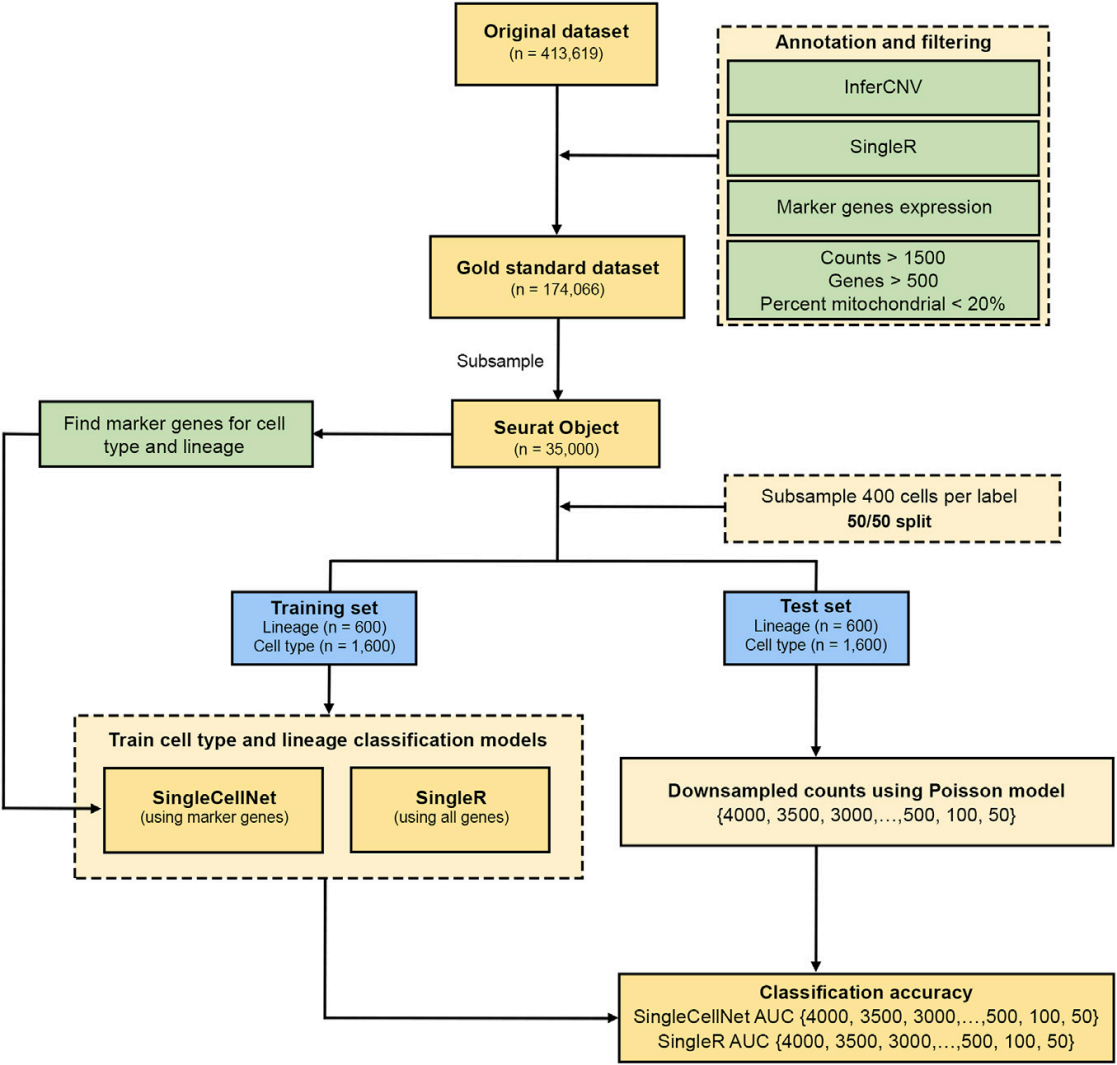
Analysis plan workflow. Flow chart shows the process of initial QC and generation of gold standard cell type annotations from the FELINE dataset. This is followed by a 50/50 split of a subsample into training and test sets for both SingleR and SingleCellNet classifiers for all datasets. The test set counts were then transformed using a Poisson model using different thresholds which is then used to determine the classification accuracy of lineage and cell type labels.

The analysis pipeline consists of the following main steps (Figure 1). We applied a stringent QC threshold on the FELINE dataset (raw UMIs) to filter for the high-confidence, high-quality cells. A combination of unsupervised and supervised expert-led approaches was used to generate the high-quality cell lineage and subtype labels which were used at the gold standard for downstream analysis. For each dataset, we first split it into training and test sets (50/50). Next, the training set was used to train the classification models to predict cell lineage and subtypes. The test set was then downsampled using Poisson model at different target UMI thresholds. We then assessed the accuracy of the classification models on the test set at different target UMI thresholds. The analysis steps are described in more details in the subsections below.

### Gold standard scRNA-seq dataset pre-processing

We used the FELINE clinical trial scRNA-seq dataset which spans 35 patients with ER-positive HER2-negative early stage breast cancer (Griffiths et al., 2021). The patient samples were processed using the 10X Chromium platform and sequenced using 150-bp paired-end sequencing at a median depth of 34,000 reads per cell (Griffiths et al., 2021). The reads were aligned to a reference genome (GRChg38) using Bioinformatics the ExperT SYstem and CellRanger v.3.0.2 pipelines (Chen and Chang, 2017). FeatureCounts was then used to generate a matrix of gene transcript UMIs for each cell, which we refer to as “original dataset” in this manuscript (Liao et al., 2014).

To generate the gold standard dataset, we applied a stringent QC filter which retained cells with >1,500 reads, 500—7,000 unique genes, and less than 20% mitochondrial content, as reported in the original study (Griffiths et al., 2021). After filtering out “low-quality” cells and doublets, we retained 176,644 “high-quality” cells. To generate Uniform Manifold Approximation and Projection (UMAP), we log-normalized, scaled the count matrix, and ran principal component analysis (PCA) on the 2000 highly variable genes using R package Seurat v.4.1.1 (Butler et al., 2018). We then constructed the K nearest neighbor and using Seurat’s FindNeighbor function on 10 principal components which was used to construct the UMAP. We then used SingleR to generate a preliminary cell type label for each cell using Human Primary Cell Atlas (HPCA) as a reference (Mabbott et al., 2013; Aran et al., 2019). These labels were used to annotate the clusters as either epithelial, stromal, or immune based on the most frequent cell type labels by SingleR. The SingleR labels were validated using lineage marker gene expression for epithelial cells (*KRT19, CDH1*), stromal cells (*FAP, HTRA1*), and immune cells (*PTPRC*) (Griffiths et al., 2021). SingleR cell type labels were also validated using cell type marker gene expression for macrophages (*CSF1R, CD163*), T-cells (*CD2, CD247*), B-cells (*MS4A1, IGHM*), fibroblasts (*COL5A1, FBLN1*), endothelial cells (*VWF*), pericytes (*RGS5*), and adipocytes (*CIDEA*). To identify putative cancer cell, we used InferCNV which predicts copy number alterations based on the positional gene expression intensity across all chromosomes (Korsunsky et al., 2019). We used stromal and immune cells as normal references for InferCNV and labelled epithelial cells with positive copy number alterations (CNA) profile as cancer cells (Griffiths et al., 2021). All downstream analyses excluded non-malignant epithelial cells. The raw (un-normalized) UMI count matrix of the gold standard dataset was used for model training and assessment. A random unbiased subsample of the gold standard dataset (*n* = 35,000) was used to create a Seurat object for downstream analysis. We removed cells with >15,000 reads to account for any missed doublets.

### External datasets

In addition to the FELINE dataset, we used a subset of whole blood scRNA-seq dataset (GSE163668) which we will refer to as “Combes dataset” (Combes et al., 2021). We combined 3 pooled libraries (GSM4995425, GSM4995426, GSM4995427) spanning 8 patients, removed RBCs and used the remaining cells with the authors’ cell type labels in our analysis. We also used a PeripheralBlood Mononuclear Cells (PBMC) dataset freely available from 10X Genomics which we will refer to as the “PBMC dataset” (10x Genomics, 2016). We processed this dataset as described in “Seurat-Guided Clustering Tutorial” (Hoffman et al., 2022). Cells with more than 5% mitochondrial counts or more than 2,500 genes or less than 200 genes were filtered out. After clustering the cells, cell types were annotated using the canonical markers as follows: Naive CD4^+^ T (*IL7R, CCR7*), CD14^+^ Mono (*CD14, LYZ*), Memory CD4^+^ (*IL7R, S100A4*), B cells (*MS4A1*), CD8^+^ T (*CD8A*), FCGR3A + Mono (*FCGR3A, MS4A7*), NK (*GNLY, NKG7*), DC (*FCER1A, CST3*), Platelet (PPBP).

### Low-quality cells subset

For “low-quality” cells which that were excluded from the gold standard dataset, we predicted the cell type labels using SingleR and human primary cell atlas (HPCA) as a reference (Mabbott et al., 2013; Aran et al., 2019). To generate lineage labels, we aggregated cell type predictions into lineage labels as follows: epithelial (epithelial cells), stromal (fibroblasts, endothelial cells, chondrocytes, osteoblast, smooth muscles), immune (T-cells, B-cells, macrophages, monocytes, NK cells, neutrophils). To study the outcome of the initial and optimized thresholds on cell retention rate, we combined the gold standard subsample (*n* = 35,000) with a low-quality subsample (*n* = 35,000) for a total of 70,000 cells.

### Training lineage and cell subtype classification modes

We used two different multi-class prediction algorithms for the analysis, SingleCellNet (SCN) and SingleR. SCN is a Random Forest classifier developed for scRNA-seq datasets and implemented as R package singleCellNet v.0.1.0 (Tan and Cahan, 2019). SingleR is a reference-based cell type classifier where after an internal marker genes identification step, cell identity is determined by Spearman correlation between the expression profile of the unknown cell and the reference samples e.g., HPCA (Aran et al., 2019). Due to the infeasibility to train a random forest classifier on all genes, we applied Seurat’s FindAllMarkers function (test.use = “negbinom”, min.pct = 0.5, max.cells.per.ident = 2000, logfc.threshold = 0.5) to generate lineage and cell type marker gene sets. For either lineage or cell type levels, we sampled 400 cells per label using splitCommon function implemented in R package singleCellNet v.0.1.0. The lineage and cell type samples were split 1:1 into a training and test set. For the SCN classifier, the UMI matrices of both training sets were filtered for the corresponding marker gene set previously identified. The SCN classifier was trained using scn_train function (nTopGenes = 100, nRand = 50, nTrees = 1000, nTopGenePairs = 200) implemented in the singleCellNet package. In contrast, the SingleR classifier was trained on all available genes in UMI matrices without filtering using trainSingleR function implemented in the R package SingleR v.1.6.1.

### Systematic downsampling of reads and genes

To simulate reduce average reads per cell at a pre-specified threshold, we downsampled the reads from high-quality cells. We used a Poisson distribution model to calculate a transformation factor. The probabilities density function for an integer vector *x* is defined as:

$$p\left(x\right)=\frac{\lambda^{x}e^{-\lambda }}{x!}$$

where, *λ* is the point mass (Poisson rate). For each cell, we generated a vector of random deviates of length = number of genes, and *λ* = target threshold/total reads. Reads from each cell were multiplied by their transformation factor to reduce the total counts per cell to the desired threshold.

To downsample the genes of the FELINE dataset, we first converted the UMI matrix into binary expression. For cells where n> = 1, we reduced random n genes from being expressed to not expressed (1 → 0) where n is the number of genes above test threshold. Each transformed matrix was then used to assess the accuracy of classification for the corresponding threshold. In the non-binary experiments, the remaining binary matrix was converted back to a non-binary UMI matrix for assessment while in binary-experiments, both the training and downsampled matrices were binary.

### Model assessment

Using the SCN and SingleR trained models, we generate the predicted labels for all downsampled matrices using scn_predict and classifySingleR functions, respectively. We then used the true labels to calculate the Area Under Receiver Operating Characteristic Curve (AUROCC) for both models at each threshold using the R package pROC v.1.18.0.

## Results

### Cell retention rates in gold standard scRNA-seq dataset

The diversity of cell populations within the TME introduces a challenge when applying a UMI threshold across tumor samples: a stringent, high UMI threshold would remove most of the low-quality cells, but also lose important populations with low reads like immune cells. In contrast, a lenient threshold would retain the low-UMI populations, but this could also increase the noise and possibly skewing the results of the downstream analysis. In addition, the QC step is usually performed early in the analysis pipeline where biological information (cell identities) is not yet available. Thus, a biology-driven revision of QC thresholds can be easily overseen. In the FELINE dataset, we had used 1,500 reads as a threshold for low-quality cells (Figure 1) (Griffiths et al., 2021). To construct the gold standard dataset, we used InferCNV to identify cancer cells and SingleR to predict normal cell identities which were verified by marker gene expression (Supplementary Figures S1A,B).

**FIGURE 2:**
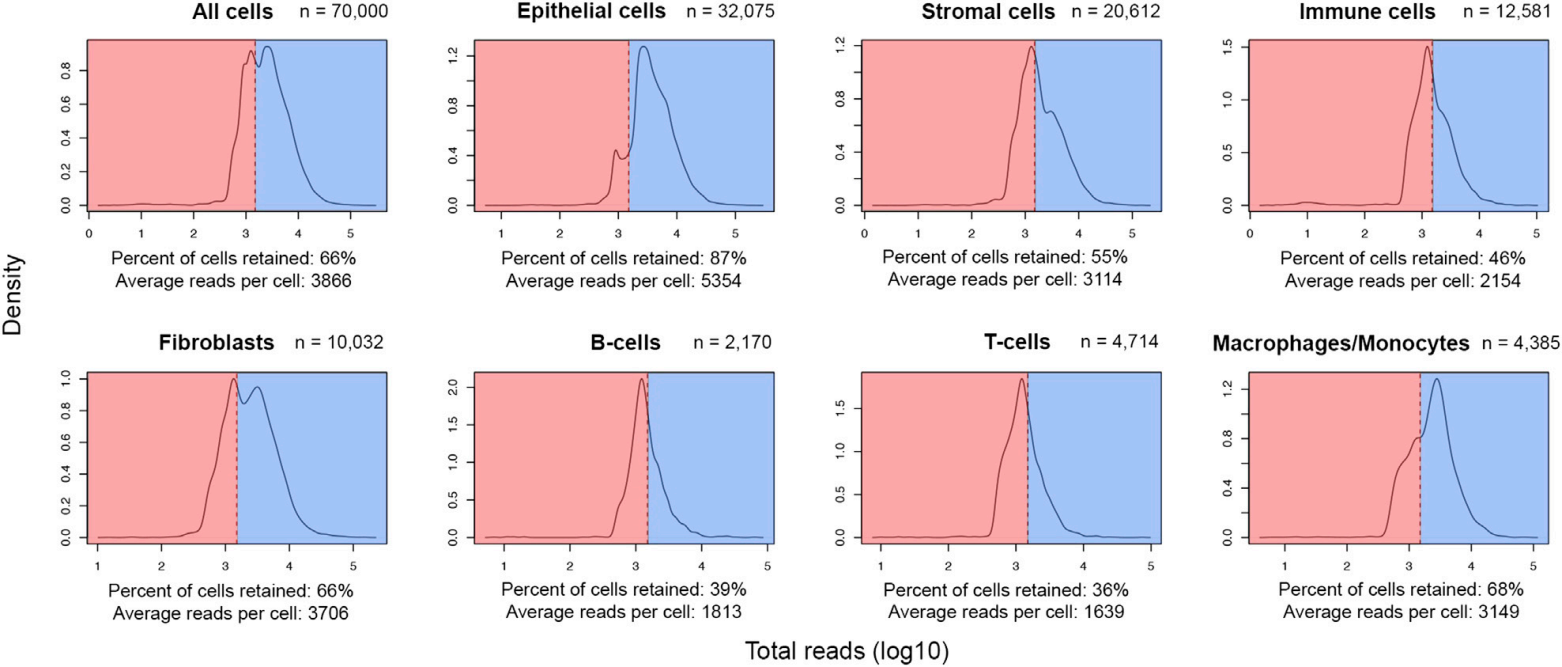
Post-QC retention rate varies across different lineages and cell types in the FELINE dataset. Density plots depict the reads-per-cell distribution across different lineages and cell types within a subsample of the original dataset (*n* = 70,000). The initial QC count cut-off (1,500 reads), as dashed line, splits the fraction of cells considered as “high-quality”, highlighted in blue, from the cells considered as “low-quality”, highlighted in red, across different cell populations. The average count and the fraction of “high-quality” cells are annotated for each population.

After meticulous cell type labelling of high-quality cells, a closer view of UMI distribution across cell lineages showed a high level of retention of epithelial cells (87%) post-QC. In contrast, only around half of the stromal and immune cells were retained (Figure 2). As breast cancer cells are of epithelial origin (Noureen et al., 2022), it is expected that actively proliferating cancer cells were driving a higher average UMI among epithelial cells (5,354 UMIs) than stromal (3,114 UMIs) or immune cells (2,154 UMIs) (Figure 2). In addition, at the finer cell subtype annotation level, two-thirds of macrophages/monocytes were retained, while only a third of the sequenced population of T and B lymphocytes were retained (Figure 2). Since B- and T-lymphocytes have the lowest average UMIs per cell in this cohort (1,813 and 1,639 respectively), the initial QC threshold only retained a small fraction of these cells for downstream analyses, suggesting an optimization of the initial threshold might be required.

### Machine learning framework guides threshold optimization and accurate classification

We developed a novel framework that systematically identified the lowest read depth threshold that can be used to accurately classify cell lineages and subtypes. Our approach trained classifiers for lineage and subtypes on a training subset of the gold standard dataset, and then predicted the cell lineage and subtypes of a held-out test or validation subset from the gold standard dataset at progressively diminished read depths. By following this approach, we could identify what is the minimum number of average reads required to accurately classify cells.

We used SCN and SingleR multi-class prediction algorithms to determine the lowest UMI threshold where sufficient biological signal was retained. We then applied a Poisson model to the test datasets to downsample to a set of desired reads threshold including 0, 50, 100, 150, 200, 250, 300, 350, 400, 450, 500, 600, 700, 800, 900, 1000, 1500, 2000, 3000 and 4000 UMIs.

**FIGURE 3:**
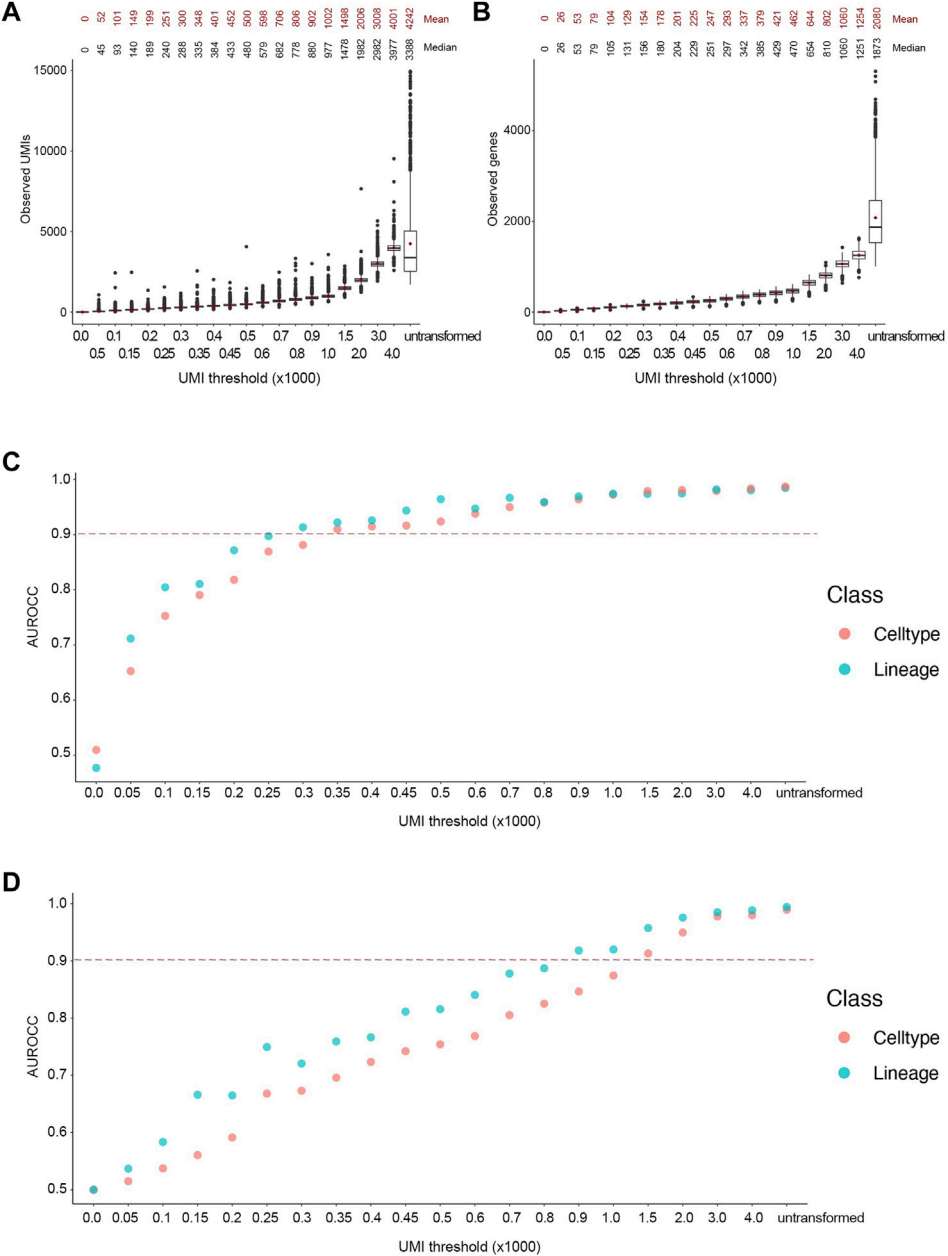
Accurate lineage and cell type classification at 450 UMIs in the FELINE dataset. **(A,B)** Boxplot showing the post-transformation distributions of observed UMIs **(A)** and number of unique genes **(B)** across all thresholds and untransformed control. Mean and median values for each distribution are denoted. **(C,D)** Area under the receiver operating characteristic curve (AUROCC) values are shown for the raw (untransformed) counts as well as the downsampled counts at different thresholds using the SingleR model **(C)** and the SingleCellNet model **(D)**. The AUROCC values for both lineage and cell type assessments are shown for each model as well as the selected AUROCC cut-off value (>0.9), dashed line.

Following the transformation, the mean number of UMIs in the downsampled cells were close to the desired UMI thresholds (Figure 3A). Indeed, the reads in the downsampled cells followed a Poisson distribution, as the variance increased at higher thresholds. Noticeably, the number of unique genes followed a Poisson distribution as well (Figure 3B). We used the trained classifiers to predict lineage and cell type labels for the downsampled cells. The ground truth and predicted labels were used to generate a confusion matrix to calculate the area under the receiver operator curve (AUROCC) at each threshold. We considered AUROCC values above 0.9 to be accurate classifications. The SingleR classifier showed an accurate prediction of both lineage and cell types at an average read depth of 450 UMIs or ∼200 genes (Figure 3C). However, the model progressively lost its predictive ability at below the 250 UMIs threshold. On the other hand, the SCN classifier showed an accurate prediction for both classes at an average read depth of 1,500 UMIs or ∼650 genes, while its predictive ability was gradually lost at thresholds below 800 UMIs (Figure 3D). The accuracy of the SingleR classifier relatively plateaued at the 350 UMI threshold. However, the accuracy of the SCN classifier increased linearly throughout with the increasing thresholds. As expected, almost all the AUROCC values for the broader lineage class were equal or higher than the narrower cell type class. It’s worth mentioning that SingleR classifier showed an overall higher classification accuracy which we attribute to the fact that SingleR calculates the spearman correlation between each cell’s expression profile and reference cells regardless of expression values while SCN only considers expressed genes e.g., non-zero expression values. Consequently, we selected the conservative 450 UMIs from the more accurate classifier at the finer cell type resolution as the optimized threshold.

In addition, we performed downsampling of gene numbers by dropping random genes at different maximum number of genes thresholds (Supplementary Figures S2A,B). Like the UMI downsampling, accurate classification (AUROCC >0.9) of lineages and cell types was achieved using 200 and 600 genes for SingleR and SCN classifier, respectively (Supplementary Figures 2C,D). We then applied the same transformation to a binary count matrix for training and test sets (Supplementary Figures S3A,B). Both classifiers yielded similar performance to non-binary counts at 250 and 450 genes for SingleR and SCN, respectively (Supplementary Figures S3C,D). Given the typical correlation between observed between UMIs and number of genes, it was not surprising that similar thresholds were obtained using the UMI-based and the gene number approaches.

### Loss of distinct clustering below the optimized threshold

**FIGURE 4:**
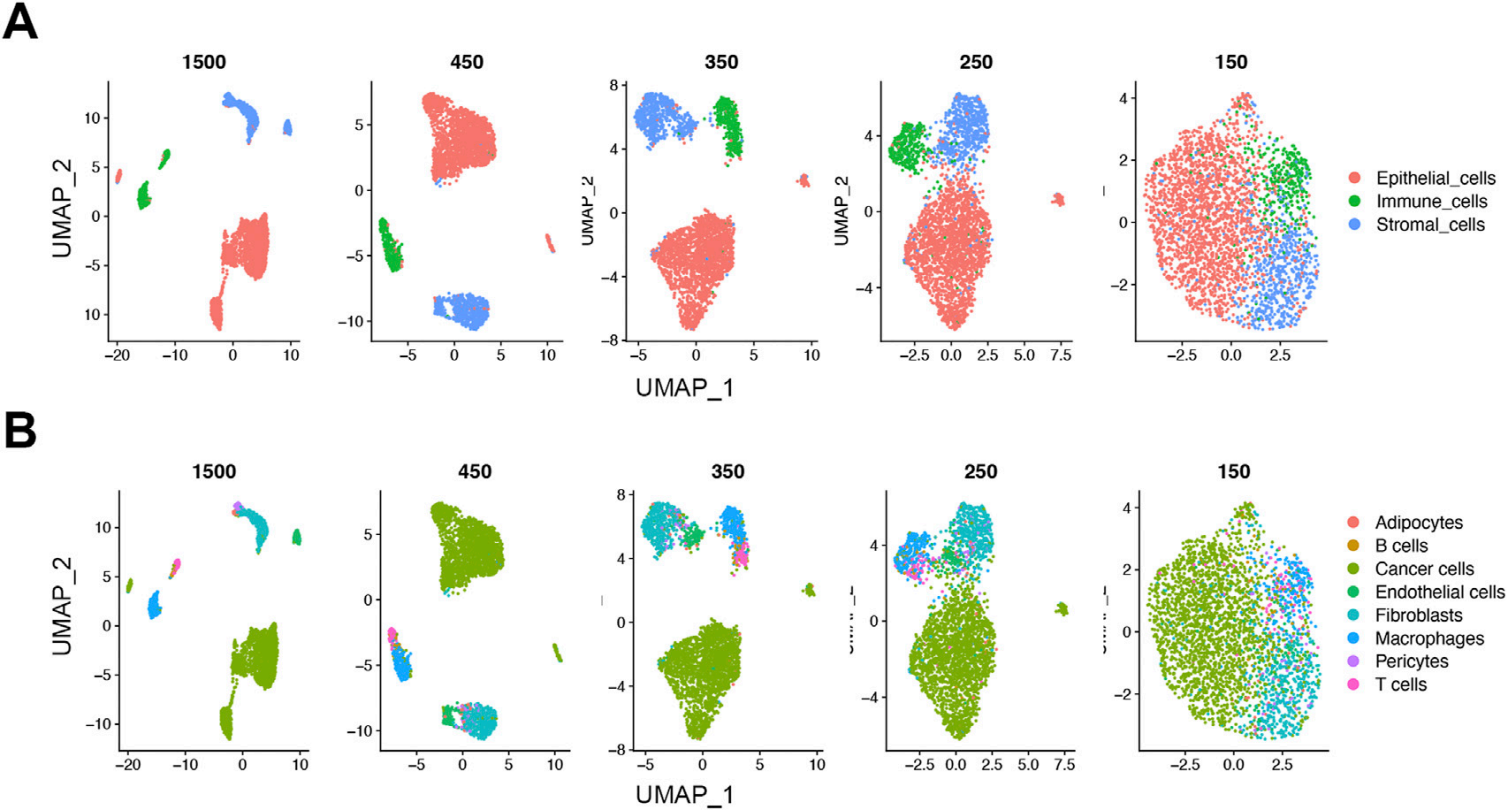
Loss of distinct cell clusters on UMAP below 450 UMIs in the FELINE dataset. Dimension reduction using Uniform Manifold Approximation and Projection (UMAP) shows that as count thresholds fall below 450 reads, a gradual loss of the distinct cell clusters is observed on lineage **(A)**, and cell type levels **(B)** (*n* = 1,500).

To see the effect of downsampling on the low dimensional data structure, we analyzed the downsampled cells from the 1500, 450, 350, 250, and 150 read thresholds using uniform manifold approximation and projections (UMAPs). Similar to the initial 1500 UMI threshold, the cells at the 450 UMI threshold showed distinct separate clusters at the lineage level (Figure 4A). As threshold was reduced, the inter-cluster distances gradually decreased. On the cell type level, the cells at the 450-threshold not only clustered by lineage but retained a rational biological hierarchy as shown by subtype cluster grouping (Figure 4B). As with the lineage level, the distinct clustering was gradually lost at lower thresholds (Figures 4A,B). This suggests that biological information retained at as low as 450 reads-per-cell maintains cell identity in our dataset.

### Optimized QC threshold rescue substantial number of cells with low transcription level

**FIGURE 5:**
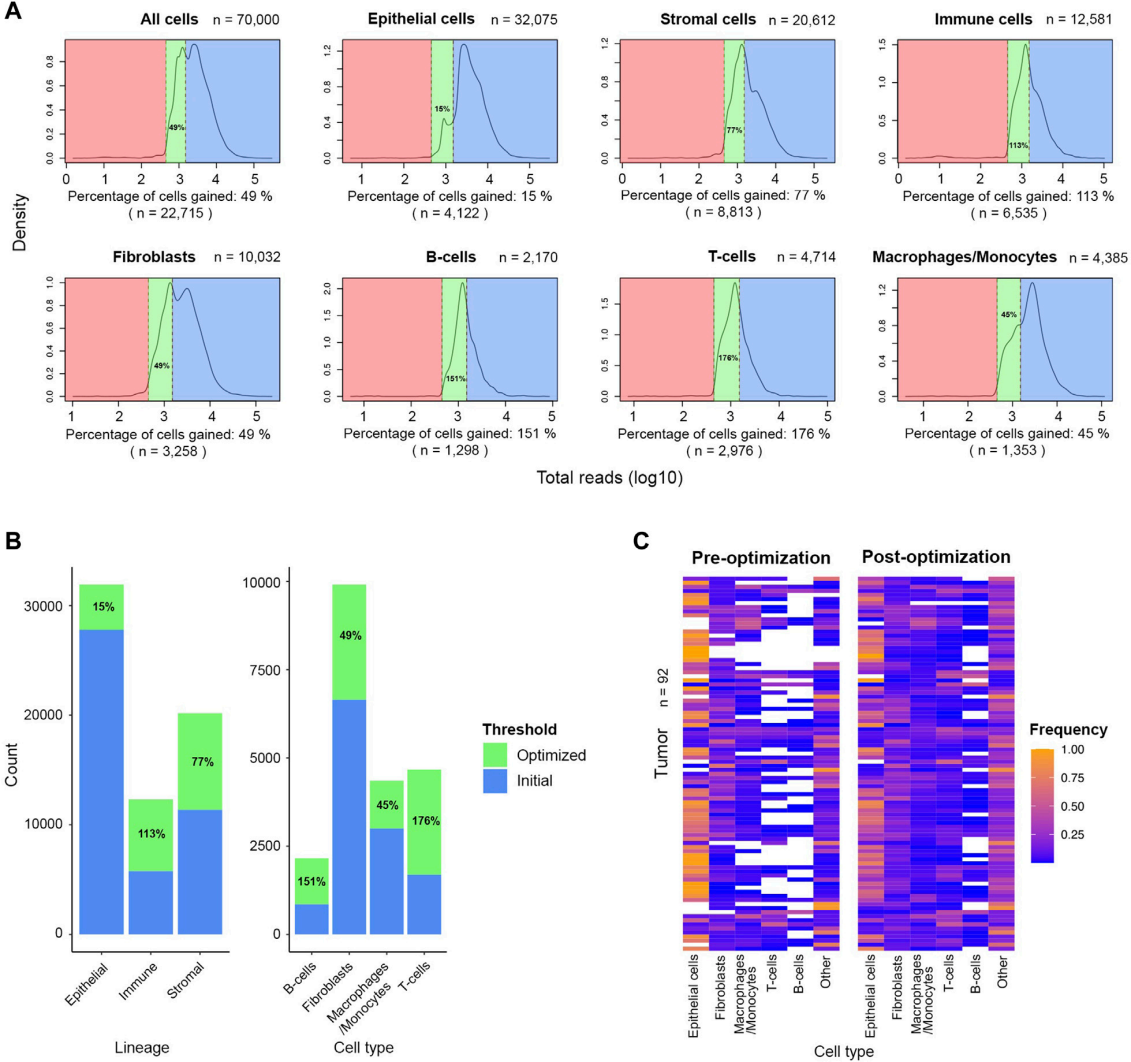
Significant number of stromal and immune cells are rescued after applying the optimized threshold of 450 UMIs in the FELINE dataset. **(A)** Density plots shows the UMI distribution across lineages and cell types within high- and low-quality cells subset (*n* = 70,000). The initial threshold (1,500 UMIs), dashed line to the right, and the optimized threshold (450 UMIs), dashed line to the left, are shown for each plot. The initial “high-quality” cells, the rescued cells after applying the revised cut-off, and the low-quality cells are highlighted in blue, green, and red, respectively. The fraction and number of cells gained relative to initially retained cells is denoted under each plot. **(B)** Bar plot showing the cell number and percentage gain for lineage and cell types after applying the optimized threshold. **(C)** Heatmap showing the relative frequency of different cell types before and after applying the optimized UMI threshold of 450 in 92 tumor samples.

To increase the number of stromal and immune cells available for downstream analysis, we applied the optimized threshold of 450 reads-per-cell to a subsample of the original dataset (*n* = 70,000). Relative to number of cells retained by the initial threshold of 1,500 reads, the optimized threshold rescued an additional 8,813 stromal cells and 6,535 immune cells, an increase of 77% and 113%, respectively (Figures 5A,B). The gain was even more prominent among the cells with low average reads as 2,976 T-cells and 1,298 B-cells were rescued which is 176% and 151%, respectively, more cells compared to the populations retained by the initial threshold. The gain among fibroblasts and macrophages/monocytes was also notable as the initial populations increased by more than 40% after applying the optimized threshold. The inclusion of rescued cells markedly improved the representation of diversity across all tumor samples, previously dominated by epithelial cells (Figure 5C). With the new thresholds, we observed a notable gain in lymphocytes across several tumors. We also noted that the optimized threshold led to the gain of 10 additional tumor samples that were excluded by the initial threshold. Thus, threshold optimization allowed the re-evaluation of cells initially penalized and discarded for their natively low expression. These rescued cells can then be incorporated in downstream analysis to characterize the TME.

### Applications in datasets containing cells with low expression and fine-grain labels

**FIGURE 6:**
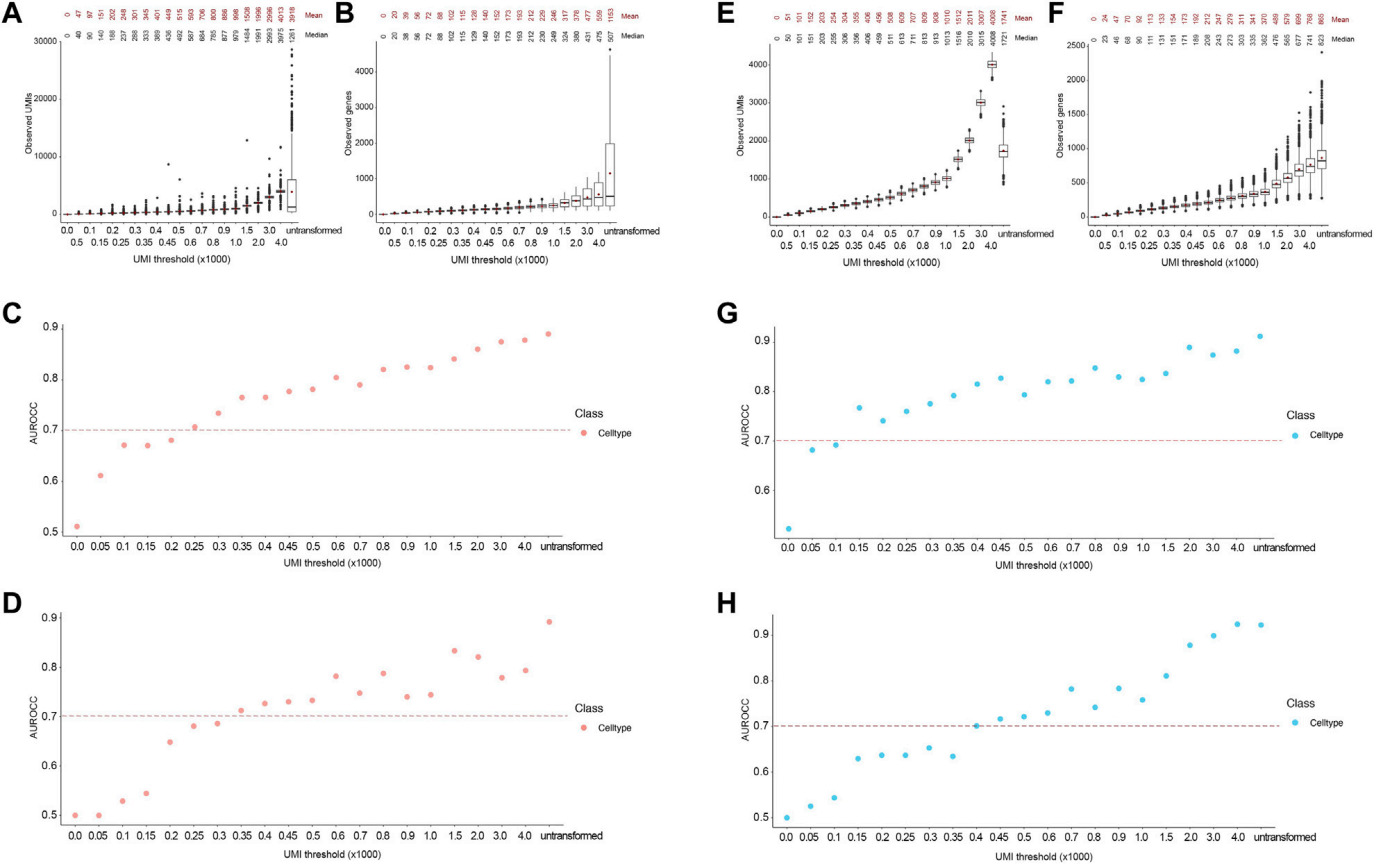
Accurate lineage and cell type classification at 250 and 150 UMIs in the Combes and PBMC datasets, respectively. For Combes dataset, **(A,B)** Boxplot showing the post-transformation distributions of observed UMIs **(A)** and number of unique genes **(B)** across all thresholds and untransformed control. Mean and median values for each distribution are denoted. **(C,D)** Area under the receiver operating characteristic curve (AUROCC) values are shown for the raw (untransformed) counts as well as the downsampled counts at different thresholds using the SingleR model **(C)** and the SingleCellNet model **(D)**. For PBMC dataset, **(E, F)** Boxplot showing the post-transformation distributions of observed UMIs **(E)** and number of unique genes **(F)** across all thresholds and untransformed control. Mean and median values for each distribution are denoted. **(G,H)** Area under the receiver operating characteristic curve (AUROCC) values are shown for the raw (untransformed) counts as well as the downsampled counts at different thresholds using the SingleR model **(G)** and the SingleCellNet model **(H)**. The AUROCC values for cell type assessment are shown for each model as well as the selected AUROCC cut-off value (>0.7), dashed line.

To test the applicability of our approach to cell types with low gene expression, we used the Combes dataset (see Methods), which contains cell types with low expression levels, including as neutrophils and platelets. As with the FELINE dataset, we applied the transformation based on Poisson distribution to systematically downsample the counts in the Combes dataset. The resultant UMI means were reflective of the desired target UMI thresholds (Figures 6A,B). Using the original published cell type labels as ground truth, the cell type classification AUROCC for the untransformed counts were about 0.9, reflecting the low average read depth of this dataset (1599 UMIs) and very low coverage in some cell types, such as neutrophils (621 UMIs) and platelets (740 UMIs). SingleR achieved AUROCC >0.7 for this dataset at 250 UMIs or ∼90 genes while SCN achieved this level of accuracy at 350 UMIs or ∼115 genes (Figures 6C,D).

Similarly, we used the 10X PBMC dataset test (see methods for details) to demonstrate that the application of the framework in cell types with fine-grain labels. The PBMC dataset (average 2371 UMIs) contains fine-grain classification of monocytes and T cells. In addition to CD14^+^ and FCGR3A + monocytes, this dataset contains different T cells subtypes like naïve CD4^+^, memory CD4^+^, and CD8^+^ T cells. Again, we applied the transformation based on Poisson distribution to systematically downsample and obtain resultant UMIs that were reflective of the desired target thresholds (Figures 6E,F). SingleR classified cells with AUROCC >0.7 at 150 UMIs or ∼70 genes threshold, while the SCN classifier achieved this level of accuracy at 400 UMIs or ∼170 genes (Figures 6G,H). Taken together, these results demonstrate that our framework can be applied to datasets containing cell types with low expression and fine granularity.

## Discussion

Single cell RNA-seq of tumor samples have proved indispensable for TME studies. This has allowed researchers to perform analyses such as in-depth classification of the composition of tumors, identifying the key signaling mechanisms operating in cancer and non-cancer cells and characterizing the heterogeneity and evolution of cancer cells, which were not previously feasible using bulk-RNA sequencing (Nath and Bild, 2021). However, the detection of rare cell populations among the diverse TME is limited by the number of cells the scRNA-seq platform can handle. The introduction of UMI-based platforms allowed for higher cell capacity which better captures the diversity of the TME. However, arbitrary UMI thresholding during the standard scRNA-seq QC risks losing considerable number of cells, such as immune cells with low expression. This can lead to inaccurate assessment of the composition of the TME and overlook critical associations between diversity and tumor traits. For example, the presence of cytotoxic T cells in the TME is strongly associated immunotherapy response in multiple cancers (Sade-Feldman et al., 2018; Kim et al., 2021; Nagasaki et al., 2022). Therefore, assessment of immune response based on diversity of infiltrating lymphocytes could improve by optimizing the UMI thresholds. Recent studies to characterize the communication networks between various individual cell types within breast tumor have revealed unique signaling networks operate in tumors resistant or sensitive to cell cycle inhibitor therapy (Griffiths et al., 2022). Resolving these communication links also requires optimizing the UMI thresholds to ensure that the TME measured using scRNA-seq reflects the true composition of the tumor.

To develop a framework that enables optimization of UMI thresholds, we used a systematic approach to downsample UMIs and accurately classify cells by lineage and cell type. We trained two classifiers, SCN and SingleR, on expert-labelled subsample of our gold standard FELINE dataset which was originally filtered using a stringent UMI threshold. We then downsampled the FELINE dataset using a Poisson transformation and evaluated the classification accuracies at various thresholds. Using a conservative AUROCC >0.9 as the cut-off for accurate classification in the FELINE dataset, we determined a significantly lower new threshold at 450 UMIs, corresponding to slightly more than 200 genes, compared to the initial threshold at 1,500 UMIs. The optimized threshold retrieved substantial number of additional cells that were initially disposed-off during filtering. The gain was prominent among cells with lower average reads than cancer cells such as stromal and immune cells. Notably, B- and T-lymphocytes populations increased more than 150% by applying the optimized threshold. We also noticed that the downsampled cells at this threshold retained similar distinct clustering patterns across lineages and cell type groups on the UMAP as the gold standard dataset. However, this was not the case at lower thresholds where the inter-cluster distances were gradually lost. We also explored gene downsampling using random gene removal at different thresholds using binary and non-binary input which resulted in similar optimal threshold to the UMI downsampling.

We further extend the application of our framework to two additional datasets. Analyses with the Combes dataset revealed that cells with low average expression, like neutrophils, can also be used in our framework to optimize thresholds. Similarly, analyses with the PBMC dataset showed that fine grain classification of cells can be accommodated in the framework.

While this approach improved the diversity of major lineages and cell types of the FELINE, Combes and PBMC datasets, its current application depends on the original labeling accuracy for cell identities. This can be challenging for some cell populations, such as cells that lack established RNA markers. Currently, the framework relies on reliable labeling of cell types in the high-quality cells. A future addition to this framework could integrate additional biological information such as pathway level information and molecular signatures to identify biologically relevant clusters and improve classification accuracy.

Our machine learning framework provides a systematic approach to optimize the initial UMI/reads threshold commonly used in scRNA-seq pipelines based on cell type annotations of cells with high read depth. This is especially valuable in rescuing cells with natively low expression like immune cells. Optimizing the QC reads threshold significantly improves the efficiency of cell diversity TME studies while maintaining accurate classification of lineage and cell type. Notably, this framework can be applied to any scRNA-seq dataset where rescuing rare or low expression cells is crucial for downstream analysis.

## Data Availability

The Combes et al. data are available through Gene Expression Omnibus under accession code GSE163668. The PBMC data are available at https://www.10xgenomics.com/resources/datasets. Other datasets and code used in this analysis are available on our GitHub repository at https://github.com/ibishara/scRNA-seq_threshold_optimization.
