# Supplementary figures and images for: A machine learning framework for scRNA-seq UMI threshold optimization and accurate classification of cell types
Source: Front Genet. 2022 Nov 25;13:982019. doi: 10.3389/fgene.2022.982019 (PMC9732024; doi:10.3389/fgene.2022.982019)

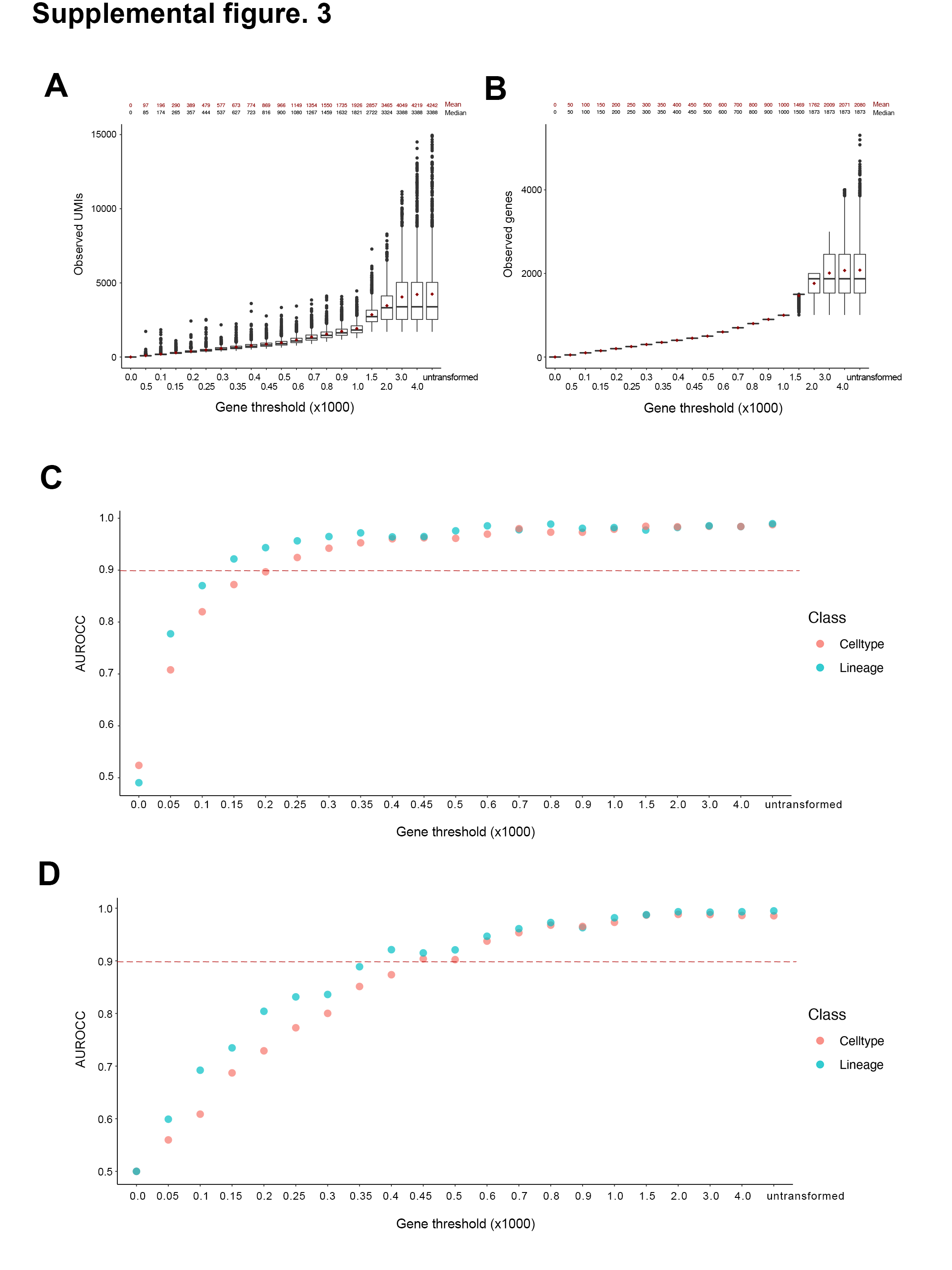

Supplement: Supplementary file 1 [file Image3.TIF]

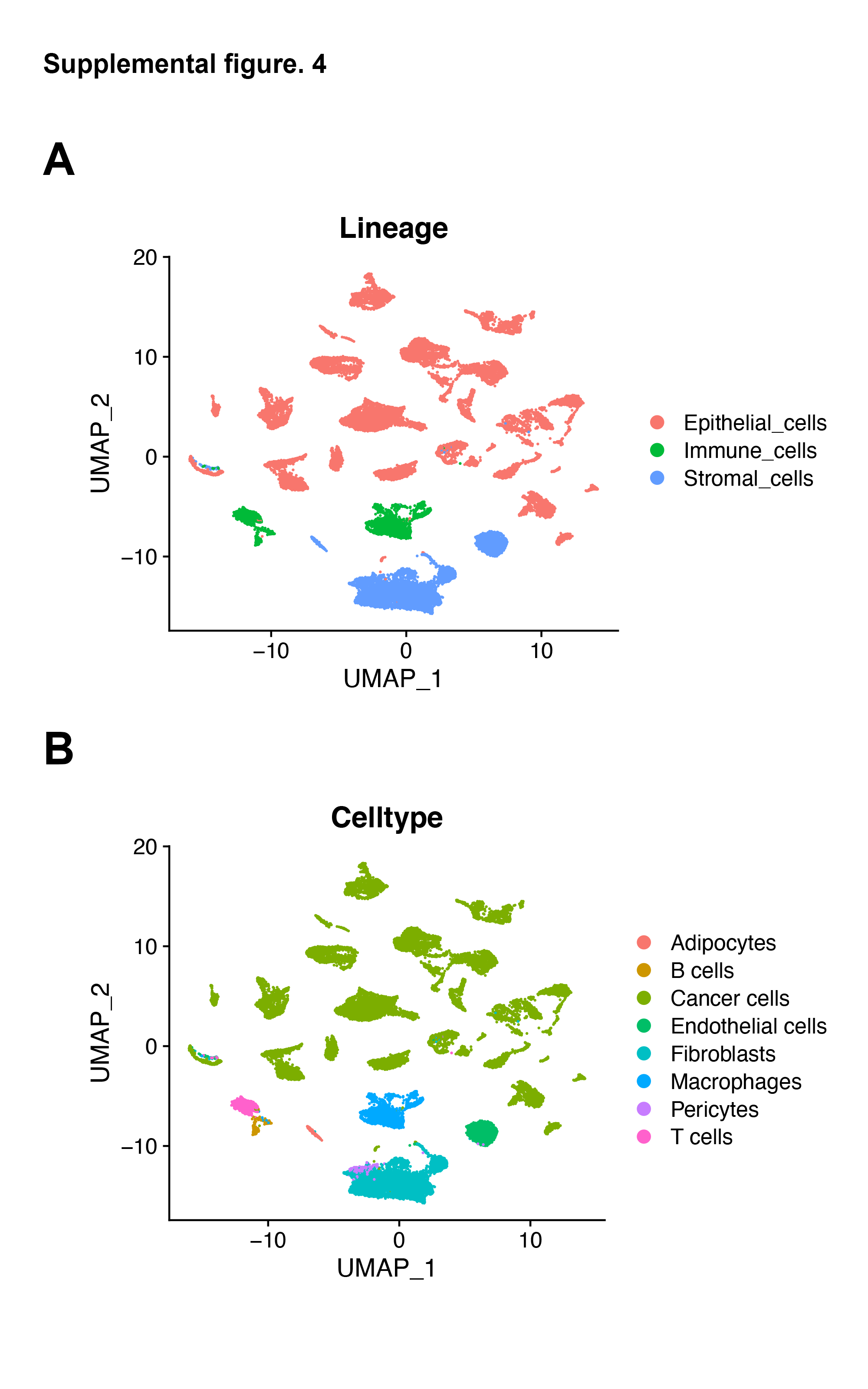

Supplement: Supplementary file 2 [file Image4.TIF]

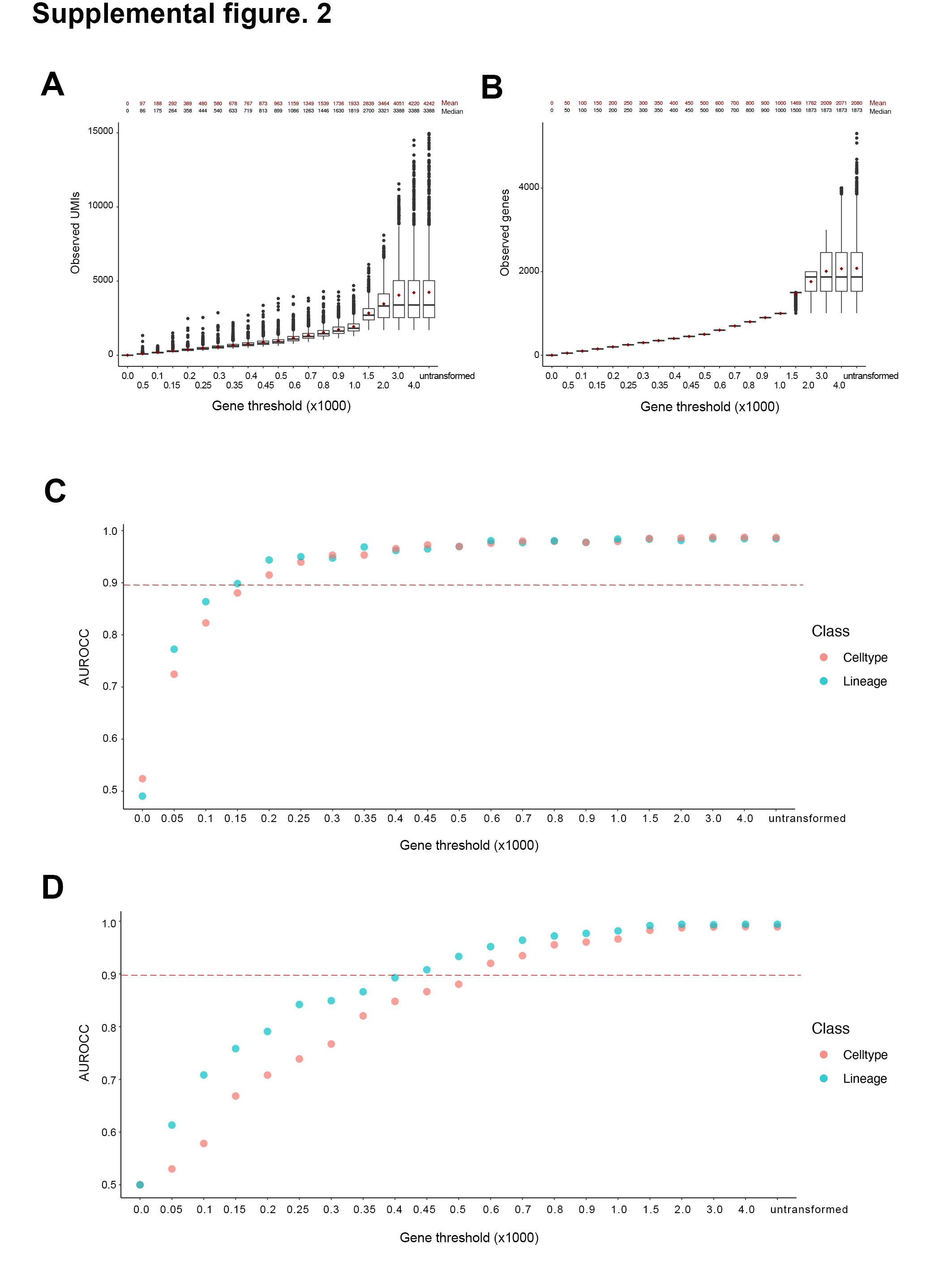

Supplement: Supplementary file 3 [file Image2.TIF]

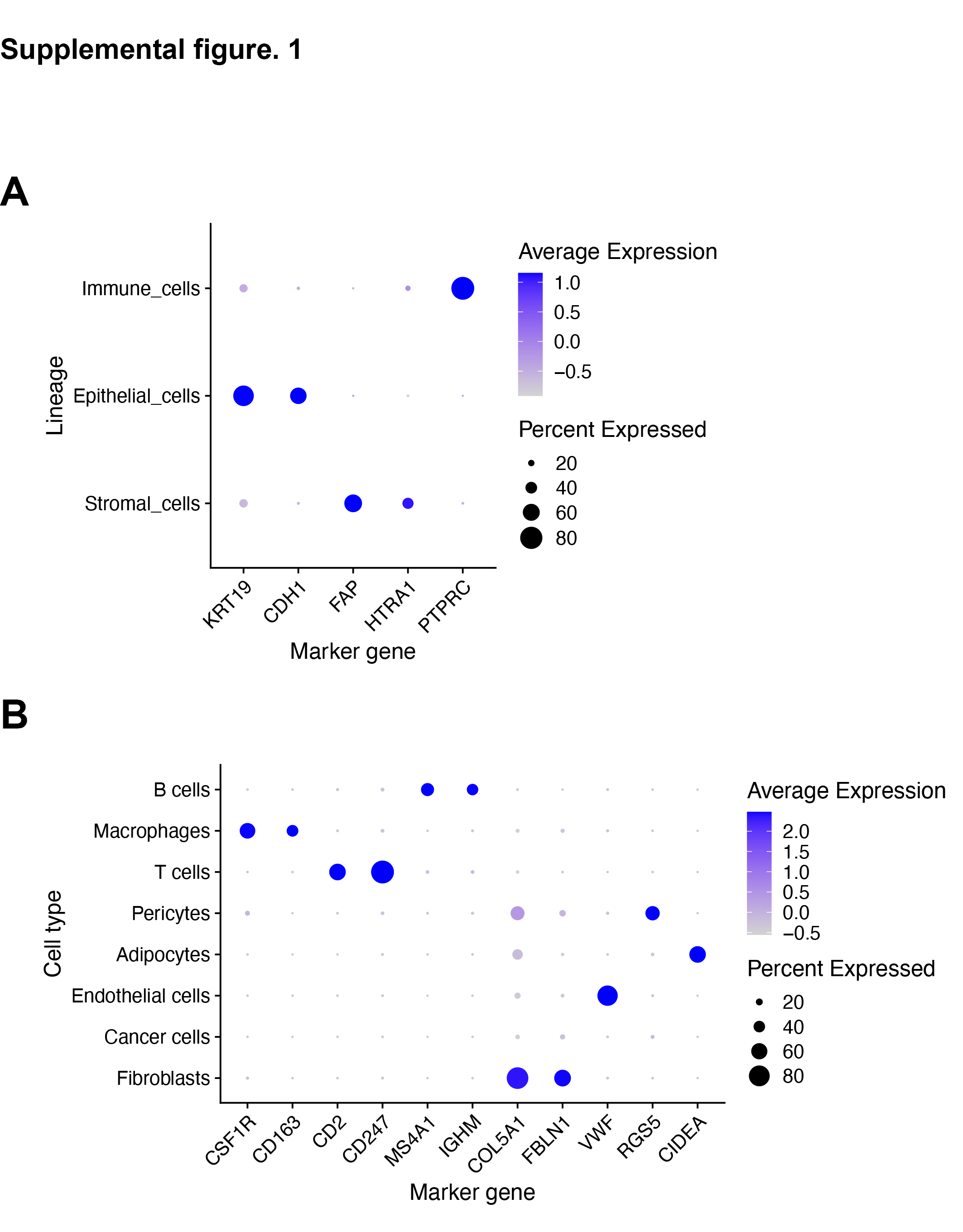

Supplement: Supplementary file 4 [file Image1.TIF]
